# STAT3 Decoy Oligodeoxynucleotides Suppress Liver Inflammation and Fibrosis in Liver Cancer Cells and a DDC-Induced Liver Injury Mouse Model

**DOI:** 10.3390/molecules29030593

**Published:** 2024-01-25

**Authors:** Hye Jin Choi, Young-Ah Kim, Junghwa Ryu, Kwan-Kyu Park, Sun-Jae Lee, Byung Seok Kim, Jeong-En Song, Joo Dong Kim

**Affiliations:** 1 Department of Surgery, School of Medicine, Daegu Catholic University, Daegu 42472, Republic of Korea; chlgpwls3210@naver.com; 2 Seoul Clinical Laboratories of Daegu, Daegu 41238, Republic of Korea; 3 Department of Radiology, School of Medicine, Daegu Catholic University, Daegu 42472, Republic of Korea; r-jung-hwa@hanmail.net; 4 Department of Pathology, School of Medicine, Daegu Catholic University, Daegu 42472, Republic of Korea; kkpark@cu.ac.kr (K.-K.P.);; 5 Department of Internal Medicine, School of Medicine, Daegu Catholic University, Daegu 42472, Republic of Korea; kbs9225@cu.ac.kr (B.S.K.);

**Keywords:** STAT3, decoy oligodeoxynucleotides, liver fibrosis, liver inflammation, bile duct proliferation, DDC-induced liver injury mouse model

## Abstract

Liver damage caused by various factors results in fibrosis and inflammation, leading to cirrhosis and cancer. Fibrosis results in the accumulation of extracellular matrix components. The role of STAT proteins in mediating liver inflammation and fibrosis has been well documented; however, approved therapies targeting STAT3 inhibition against liver disease are lacking. This study investigated the anti-fibrotic and anti-inflammatory effects of STAT3 decoy oligodeoxynucleotides (ODN) in hepatocytes and liver fibrosis mouse models. STAT3 decoy ODN were delivered into cells using liposomes and hydrodynamic tail vein injection into 3,5-diethoxycarbonyl-1,4-dihydrocollidine (DDC)-fed mice in which liver injury was induced. STAT3 target gene expression changes were verified using qPCR and Western blotting. Liver tissue fibrosis and bile duct proliferation were assessed in animal experiments using staining techniques, and macrophage and inflammatory cytokine distribution was verified using immunohistochemistry. STAT3 decoy ODN reduced fibrosis and inflammatory factors in liver cancer cell lines and DDC-induced liver injury mouse model. These results suggest that STAT3 decoy ODN may effectively treat liver fibrosis and must be clinically investigated.

## 1. Introduction

Developing treatment strategies to prevent liver fibrosis-induced cirrhosis and its associated complications is essential [1,2]. Liver fibrosis is caused by an imbalance between extracellular matrix (ECM) protein synthesis and degradation; excessive deposition changes liver structure and function [3]. Hepatocytes process and absorb nutrients, produce serum proteins and blood clotting factors, and metabolize pharmaceutical drugs and toxins. Cholangiocytes are biliary epithelial cells that form bile duct tubules responsible for bile modification and transportation along the biliary tree into the small intestine. The nonparenchymal fraction comprises endothelial cells, vascular smooth muscle cells, hepatic stellate cells (HSCs), portal fibroblasts, and diverse immune cells, which can affect liver regeneration [4].

HSCs are nonparenchymal perisinusoidal cells in the subendothelial space between hepatocytes and sinusoidal endothelial cells [5,6]. HSCs are the primary cell type responsible for excess ECM deposition during fibrogenesis [7]. Transforming growth factor β (TGF-β) is a major profibrogenic cytokine and a potent collagen production inducer [8,9]. TGF-β overexpression is associated with liver fibrosis in diverse animal models [10] and patients with chronic liver diseases [11]. Expression of the Snail family transcriptional repressor 1 (Snail) has been demonstrated in fibrotic liver cholangiocytes and hepatocytes and is a central transcription factor in HSC activation, demonstrating its essential role in regulating liver fibrosis [12]. Tumor necrosis factor α (TNF-α) also profoundly affects hepatocyte differentiation. TNF-α strongly enhances bile ductular hepatocyte transdifferentiation within a collagen-rich matrix, particularly by suppressing hepatocyte differentiation and enhancing ductular morphogenesis. Direct effects of TNF-α on hepatocyte differentiation may be involved in the pathogenesis of hepatic dysfunction associated with fibrosis in chronic liver injury linked to fibrosis [13].

The JAK-STAT pathway was identified in the early 1990s as a key signaling cascade mediating cytokine receptor-derived signaling in mammals [14,15]. Of the seven known members of the mammalian STAT family, STAT3 functions as a crucial oncogenic signaling mediator [16,17,18]. Several strategies have been developed to inactivate STAT3, such as using aptamers and peptidomimetics to target STAT3 proteins and antisense oligonucleotides to suppress STAT3 expression [19,20,21,22]. Moreover, STAT3 promotes immunosuppression in the tumor microenvironment [23]. In the liver, the JAK-STAT pathway, a diverse array of cytokines, and other mediators, including growth factors and viral proteins, are activated by growth hormones [24,25,26]. Phosphorylated STAT3 is more aggressive against liver cancer [27]. However, no therapies targeting STAT3 inhibition against liver disease have been approved to date.

Because transcription factors bind to specific DNA sequences [28], short-dissipated oligodeoxynucleotides (ODN) can also be used as probes and studied using electrophoretic mobility shift assays. ODN, with a binding sequence for specific transcription factors, have also been used to regulate gene expression in living cells. This strategy involves intracellular delivery of such “decoy” ODN, which are then recognized and bound by the target factor. The decoy occupies the DNA-binding site of the transcription factor, rendering the protein incapable of binding to the promoter region of the target gene [28]. Decoys were first described as tools for investigating transcription factor activity in cell culture systems [29]. Using decoy ODN for therapeutic manipulation of gene expression was first described in 1995 [30]. Furthermore, modifying the ODN composition to prolong ODN stability in vivo and developing a delivery system for liver tissues are critical for enhancing potential therapeutic efficacy.

In 3,5-diethoxycarbonyl-1,4-dihydrocollidine (DDC)-induced liver fibrosis mouse model, protoporphyrin accumulation obstructs the hepatobiliary system, leading to biliary damage and resulting in highly proliferative cells forming bile duct-like structures that remain restricted to the portal mesenchyme, delineated by a thin laminin layer, and exhibiting dense portal inflammation [31]. Protoporphyrin first accumulates in the cytoplasm of parenchymal and Kupffer cells. Because of its hydrophobic nature, excess protoporphyrin can only exit the liver through biliary excretion, leading to the deposition of this poorly soluble molecule in bile canaliculi and ducts, forming crystals that increase in size and number [31,32]. Therefore, DDC-induced liver damage in mice has been used to investigate the pathogenesis and treatment of liver fibrosis. 

This study aimed to investigate the anti-fibrotic and anti-inflammatory effects of STAT3 decoy ODN in hepatocytes and liver fibrosis mouse models. 

## 2. Results

### 2.1. STAT3 Decoy ODN Inhibit the Proliferation of Hepatic Cancer HuH-7 and HepG2 Cells

**Figure 1:**
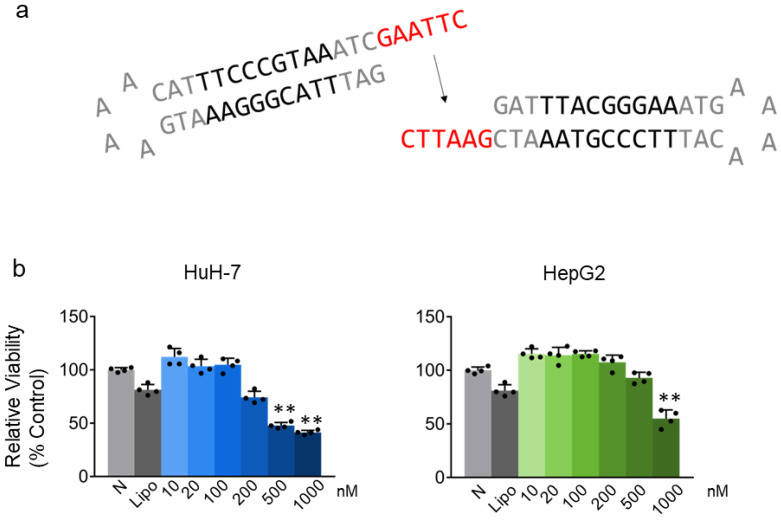
Effect of STAT3 decoy oligodeoxynucleotides (ODN) on cell viability as determined through the CCK-8 assay. Schematic diagram of STAT3 decoy ODN for humans (**a**). Cell viability of HuH-7 and HepG2 cells measured 24 h after STAT3 decoy ODN treatment. Gray bars indicate the control group, N: Naïve, Lipo: Lipofectamine concentration used to transfect cells with STAT3 decoy ODN. Blue or green bars indicate STAT3 decoy ODN treatment at 10, 20, 100, 200, 500, and 1000 nM concentration (**b**). Data are expressed as the mean ± standard deviation (*n* = 4). ** *p* < 0.01 vs. Lipo group. Statistical analysis was conducted using Student’s *t*-test. The black dots in the bar graph represent the individual values for each sample.

We investigated the effect of STAT3 decoy ODN on hepatic cancer cell proliferation. HuH-7 and HepG2 cells were incubated for 24 h after treatment with 10, 20, 100, 200, 500, and 1000 nM STAT3 decoy ODN. STAT3 decoy ODN inhibited hepatic cancer cell proliferation in a dose-dependent manner, and the inhibitory effect was significant at a concentration of 500 nM in HuH-7 cells and 1000 nM in HepG2 cells when compared to the maximum concentration of Lipofectamine (*p* < 0.01; Figure 1b).

### 2.2. STAT3 Decoy ODN Suppress STAT3 Target Genes in HuH-7 Cells

**Figure 2:**
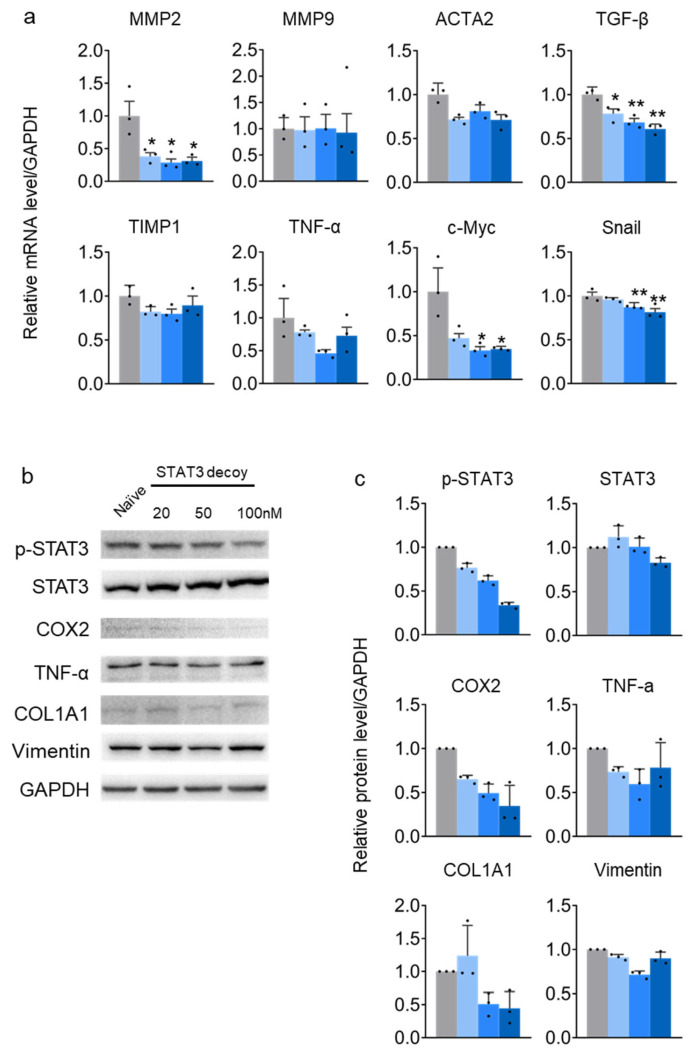
STAT3 decoy ODN suppress STAT3 transcriptional activity in HuH-7 cells. The gray bar indicates the naïve group, and the blue bars indicate STAT3 decoy ODN treatment at 20, 80, and 160 nM for 24 h. The expression of target genes is normalized to that of *GAPDH*. Error bars indicate the mean ± standard error of the mean (*n* = 3). * *p* < 0.05, ** *p* < 0.01 vs naïve cells. Statistical analysis was conducted using Student’s *t*-test (**a**). The gray bar indicates the naïve group, and the blue bars indicate STAT3 decoy ODN treatment at 20, 50, and 100 nM for 24 h. Proteins were detected using Western blotting (**b**). Protein levels in different groups (**c**). The black dots in the bar graph represent the individual values for each sample.

In HuH-7 cells, the expression of STAT3 target genes, namely, matrix metalloproteinase 2 (*MMP2*), actin alpha 2 (*ACTA2*), transforming growth factor beta (*TGF-β*), tumor necrosis factor (*TNF-α*), *c-Myc*, and snail family transcriptional repressor 1 (*SNAIL*), were suppressed by STAT3 decoy ODN. Tissue inhibitors of metalloproteinase 1 (*TIMP1*) and *MMP9* genes, suppressed by STAT3, were not altered by STAT3 decoy ODN (Figure 2a). Furthermore, STAT3 decoy ODN may reduce liver fibrosis and inflammation by inhibiting genes that increase liver fibrosis and promoting those that suppress liver fibrosis in hepatic cancer cells. Therefore, cells were transfected with the decoy for 24 h to evaluate the anti-inflammatory effect of STAT3 decoy ODN on HuH-7 cells. STAT3 decoy ODN treatment successfully decreased p-STAT3 formation dose-dependently without altering STAT3 protein expression. The level of COX2 protein, an NF-κB target protein, was measured to verify the decrease in NF-κB expression, an inflammatory marker. COX2 was decreased after STAT3 decoy ODN treatment in a dose-dependent manner, which is predicted to reduce inflammation. 

Furthermore, TGF-β expression was measured to verify the anti-fibrotic effect of STAT3 decoy ODN. STAT3 decoy ODN decreased TGF-β expression at a maximum concentration of 50 nM. TNF-α and IFN-γ expression levels were measured to verify the anti-inflammatory effect of STAT3 decoy ODN. INF-γ levels decreased at a dose of 50 nM but increased at 20 nM compared to the naïve cells. STAT3 decoy ODN did not alter TNF-α expression, verifying that the anti-inflammatory effect was challenging (Figure 2b,c).

### 2.3. STAT3 Decoy ODN Suppress STAT3 Target Genes in HepG2 Cells

**Figure 3:**
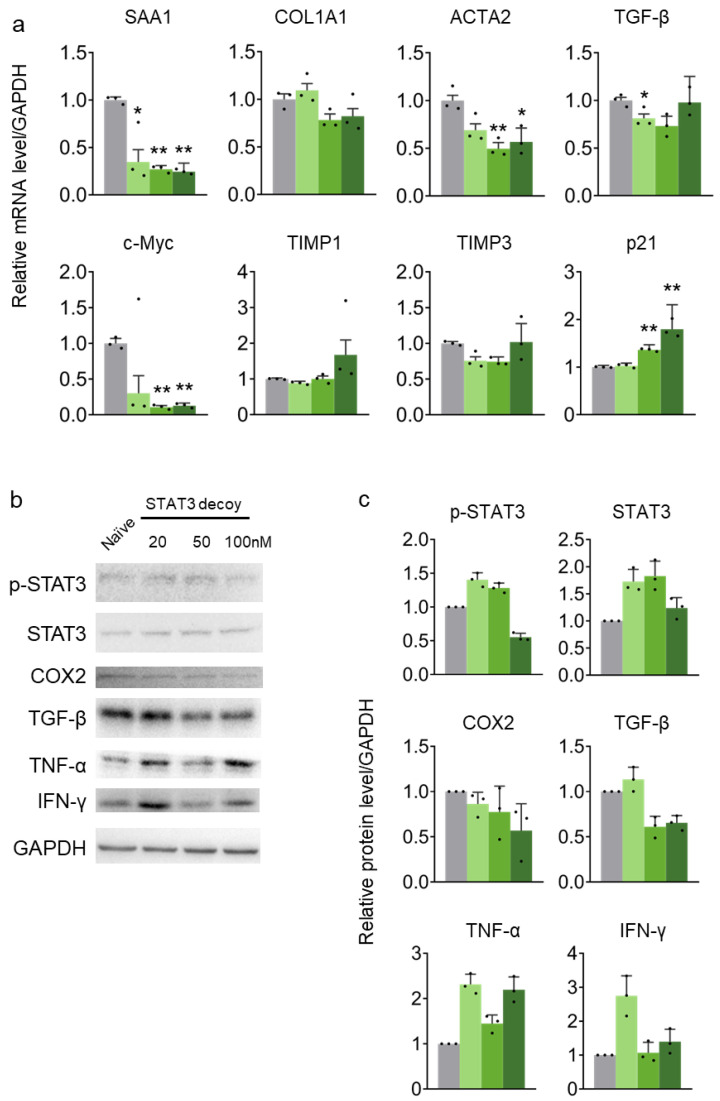
STAT3 decoy ODN suppress STAT3 transcriptional activity in HepG2 cells. The gray bar indicates the naïve group, and the green bars indicate STAT3 decoy ODN treatment at 20, 80, and 160 nM for 24 h. The expression of target genes is normalized to that of *GAPDH*. Error bars indicate the mean ± standard error of the mean (*n* = 3). * *p* < 0.05, ** *p* < 0.01 vs. naïve cells. Statistical analysis was conducted using Student’s *t*-test (**a**). Proteins were detected using Western blotting (**b**). The expression levels of proteins were quantified using Image J software (version 2.3.0). The gray bar indicates the naïve group, and the blue bars indicate STAT3 decoy ODN treatment at 20, 50, and 100 nM for 24 h (**c**). The black dots in the bar graph represent the individual values for each sample.

In HepG2 cells, the expression of STAT3 target genes, namely, serum amyloid A protein 1 (SAA1), collagen type I alpha 1 chain (COL1A1), ACTA2, TGFB, and c-Myc were suppressed by STAT3 decoy ODN. STAT3 decoy ODN increased the expression of genes suppressed by STAT3, namely, TIMP1, 3, and cancer suppressor p21. STAT3 decoy ODN effectively served as a competitive DNA binder, inhibiting STAT3 target gene expression (Figure 3a). Similar to the results in HuH-7 cells, p-STAT3 was decreased by STAT3 decoy ODN in HepG2 cells, regardless of STAT3 expression. COX2 was also decreased after STAT3 decoy ODN treatment dose-dependently, similar to the HuH-7 cell results. TNF-α decreased after STAT3 decoy ODN treatment; however, treatment was ineffective at the highest dose. COL1A1 and Vimentin protein expression levels were measured to verify the anti-fibrotic effect of STAT3 decoy ODN. STAT3 decoy ODN decreased COL1A1 levels in a dose-dependent manner, whereas Vimentin level was decreased only at the highest dose (Figure 3b,c). 

### 2.4. Treatment with Decoy ODN in DDC-Induced Liver Fibrosis Mice

**Figure 4:**
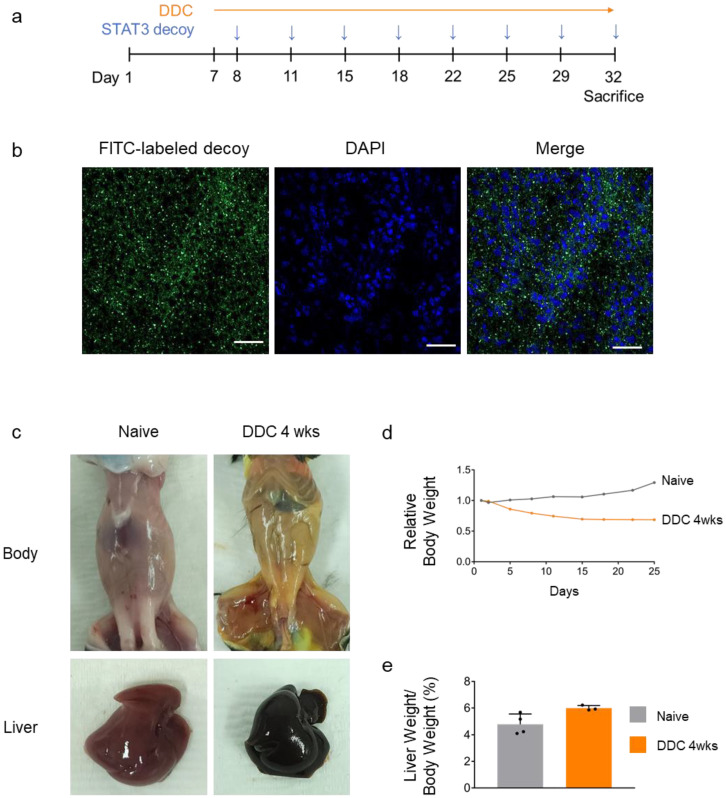
Transfection efficiency of decoy ODN and establishment of a 3,5-diethoxycarbonyl-1,4-dihydrocollidine (DDC)-induced liver injury mouse model. Schematic diagram of STAT3 decoy ODN for mouse (**a**). Verification of fluorescein (FITC)-labeled STAT3 decoy ODN in naïve mouse liver. Fluorescence activity was detected in the cytoplasm and nucleus, indicating FITC-labeled STAT3 decoy ODN deposition. Original scale bar = 50 μm (**b**). Changes in the skin color and liver of mice fed 0.1% DDC diet for four weeks (**c**). The body weight of DDC-fed mice was lower than that of naïve mice (**d**). The liver weight/body weight ratio of DDC-fed mice was greater than that of naïve mice (**e**). The black dots in the bar graph represent the individual values for each sample.

To further evaluate the anti-fibrotic efficacy of STAT3 decoy ODN in vivo, DDC-induced fibrosis mouse model was treated with fluorescein (FITC)-labeled STAT3 decoy ODN (STAT3 decoy ODN-treated mice with 0.1% DDC-supplemented diet (DDC + STAT3 mice)) (Figure 4a). To verify the effective transfer of STAT3 decoy ODN in vivo, we measured the distribution of FITC-labeled STAT3 decoy ODN in the mouse liver using confocal microscopy. FITC-labeled STAT3 decoy ODN exhibited strong fluorescence in the mouse liver cytoplasm (Figure 4b). 

The liver of vehicle-treated mice with 0.1% DDC-supplemented diet (DDC + Veh mice) turned black, and the surface became rough (Figure 4c). The body weight of DDC + Veh mice was lower than that of untreated (naïve) mice (Figure 4d). The liver weight/body weight ratio of DDC + Veh mice was higher than that of naïve mice (Figure 4e). Jaundice was observed in mice after four weeks. These findings confirmed the establishment of a DDC-induced liver fibrosis mouse model. 

### 2.5. STAT3 Decoy ODN Ameliorate DDC-Induced Liver Fibrosis

**Figure 5:**
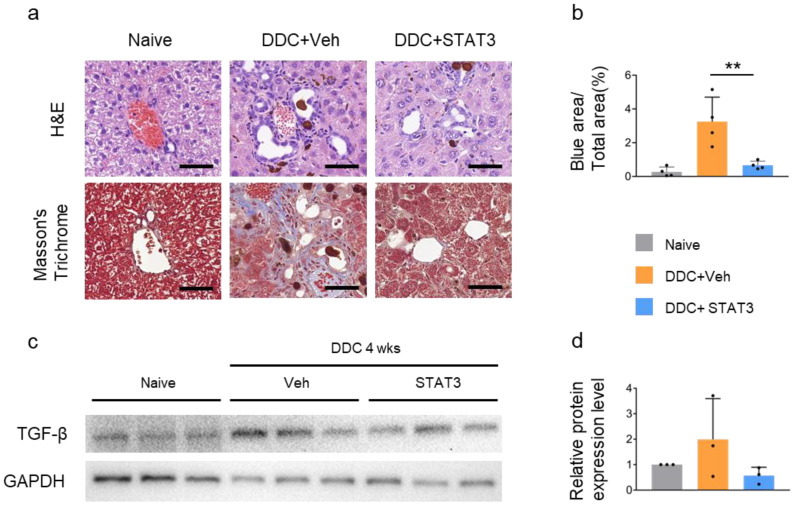
STAT3 decoy ODN suppress hepatic fibrogenesis. Mice were not challenged or challenged with DDC-fed + Vehicle IV injection (DDC + Veh) or DDC-fed+STAT3 decoy ODN IV injection (DDC + STAT3). Morphometric analysis of fibrotic lesions in the liver. Original scale bar = 50 μm (**a**). Data are expressed as the mean ± standard deviation (*n* = 4). A one-way ANOVA was conducted to compare the means of the three groups (*p* < 0.01) ** *p* < 0.01. Statistical analysis was conducted using Student’s *t*-test (**b**). Representative Western blotting image showing TGF-β expression (**c**). The expression level of TGF-β was quantified using Image J software (version 2.3.0). The results are presented as mean ± standard deviation (SD). Data are normalized against *GAPDH* expression. The results of the one-way ANOVA did not show any significant difference between the means of the three groups (*p* > 0.05) (**d**). H&E, hematoxylin and eosin staining. The black dots in the bar graph represent the individual values for each sample.

Hematoxylin and eosin (H&E) staining revealed increased ductular proliferation and porphyrin plugs occluding the lumina of small bile ducts in DDC + Veh mice. Furthermore, Masson’s trichrome staining demonstrated collagen accumulation in DDC + Veh mice, whereas collagen expression decreased in DDC + STAT3 mice, suggesting that DDC-induced liver fibrosis was regulated by STAT3 decoy ODN (Figure 5a,b). HSC leads to a pro-fibrotic milieu with increased TGF-β expression. TGF-β expression was increased in the liver of DDC + Veh mice but was decreased in DDC + STAT3 mice, indicating the anti-fibrotic effect of STAT3 decoy ODN on liver fibrosis (Figure 5c,d). 

### 2.6. STAT3 Decoy ODN Ameliorat DDC-Induced Fibrosis and Inflammation

**Figure 6:**
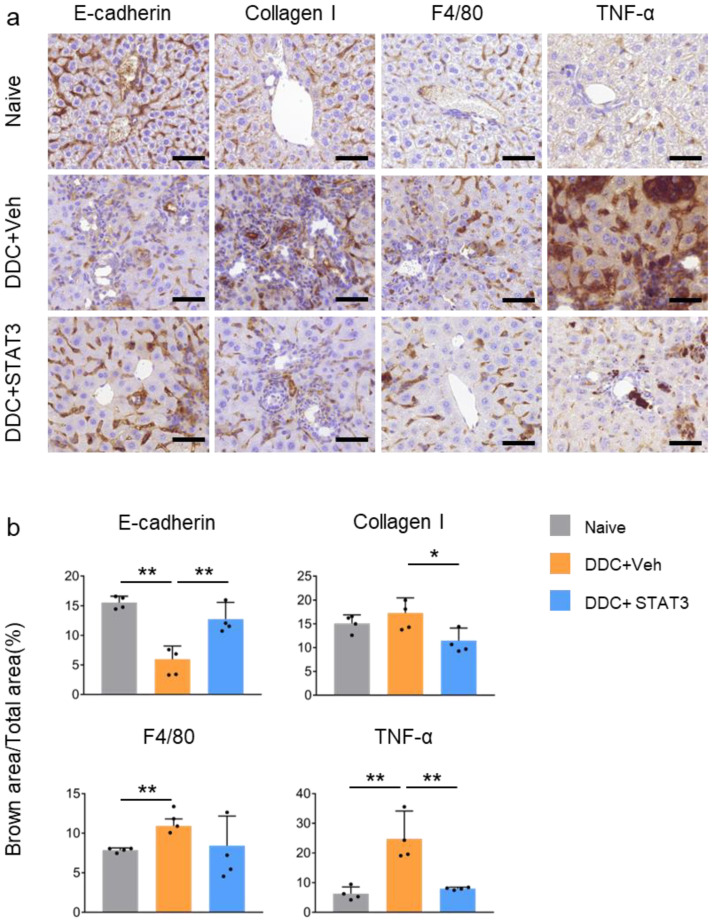
STAT3 decoy ODN ameliorat liver fibrosis and inflammation in the liver of DDC-induced liver injury mouse model. Mice were not challenged or challenged with DDC-fed + Vehicle IV injection or DDC-fed + STAT3 decoy ODN IV injection. Representative immunohistochemistry staining showing E-cadherin, Collagen I, F4/80, and TNF-α expression around the portal vein of hepatic fibrosis in mice. Original scale bar = 50 μm (**a**). Quantifying expression levels of E-cadherin, Collagen I, F4/80, and TNF-α (**b**). Data are expressed as the mean ± standard deviation (*n* = 4). The expression levels of E-cadherin, Collagen I, and TNF-α were significantly different among the three groups (*p* < 0.05), while the expression level of F4/80 was not significantly different (*p* = 0.058). * *p* < 0.05, ** *p* < 0.01. Statistical analysis was conducted using Student’s *t*-test. The black dots in the bar graph represent the individual values for each sample.

The loss of E-cadherin accompanies liver fibrosis. In line with this, DDC feeding decreased E-cadherin expression and was almost completely ameliorated by STAT3 decoy ODN treatment. Following liver injury, HSCs undergo phenotypic changes that increase the deposition of ECM proteins, including type I collagen, in the hepatic sinusoid. Collagen I level was increased in DDC-fed mice but decreased in DDC + STAT3 mice. Moreover, F4/80 immunohistochemistry (IHC) revealed that macrophage accumulation increased significantly in the liver of mice fed DDC and decreased after STAT3 decoy ODN treatment. The increased TNF-α expression in DDC-fed mice was markedly reduced after treatment with STAT3 decoy ODN. The decrease in F4/80 and TNF-α expression verified that inflammation increased by DDC-induced liver injury was rescued by STAT3 decoy ODN (Figure 6). 

## 3. Discussion

We investigated the anti-inflammatory and anti-fibrotic effects of STAT3 decoy ODN treatment in vitro and in vivo. MMPs are controlled by TIMPs and are essential for tissue remodeling, fibrosis, and inflammation. MMP2 levels increase during liver injury and decrease after treatment or in the normal state [33]. MMP2 expression was decreased in HuH-7 cells, but TIMP1 expression was not increased. This suggests that MMP2 suppression may occur through other mechanisms. In HepG2 cells, TIMP1 and TIMP3 increased, but MMP2 and MMP9 did not; these genes were expressed at very low concentrations and were undetected. 

Synthetic STAT3 inhibitors inhibit the proliferation, migration, and stress fiber formation in HSCs and suppress the expression of fibrosis-promoting factors α-SMA, collagen, and TIMP1 [34]. However, analyzing the hepatocyte cell line used in this study was challenging because of significantly lower expression levels of collagen and fibrous factors. COL1A1 and Vimentin levels could be measured in HepG2 cells, where they were decreased; however, they were not detected in HuH-7 cells. The expression in the liver tissue is possibly low, as hepatocytes use HSC-derived ECM proteins for liver fibrosis. In future studies, we plan to use STAT3 decoy ODN for treatment in an artificial injury state with TGF-β and TNF-α to overcome this limitation.

DDC-induced hepatic fibrosis was related to increased biliary porphyrin secretion and biliary epithelial cell activation with the development of bile duct injury, leading to pericholangitis and ductular reaction, resulting in portal-portal fibrosis [32]. Although no background staining in the liver IHC was observed, collagen increased during DDC feeding [35]. However, collagen levels did not increase significantly in DDC-induced fibrotic liver. Contrary to other findings, our experiment suggests that an unsuitable amount of DDC or treatment duration was used to increase collagen, and DDC treatment duration or concentration must be altered to observe changes in collagen [36,37].

TGF-β function is cell-dependent and may vary at different liver disease stages [38]. TGF-β is critical in controlling liver mass by regulating hepatocyte apoptosis in liver disease development and regeneration. Therefore, loss of TGF-β activation can lead to hyperproliferative disorders and cancer. High TGF-β levels, due to liver injury, activate HSCs, promote myofibroblast transdifferentiation, stimulate ECM production, and are thus a pivotal fibrogenic cytokine [39]. Furthermore, TGF-β is a novel target of STAT3-regulated genes involved in fibrogenesis [40]. We demonstrated that STAT3 decoy ODN reduced TGF-β expression, consolidating TGF-β as a STAT3 target gene and the anti-fibrogenic effect of STAT3 decoy ODN.

No effective therapy for STAT3 inhibition has been approved despite abundant evidence supporting STAT3 as a potential anticancer target. This is because STAT3 activates anti-inflammatory signals, which rescue the damaged liver [41]. The increase in STAT3 during liver damage is attributed to tissue recovery mediated by increased p21 and natural killer cells [42]. However, STAT3 decoy ODN inhibited fibrosis and inflammatory signaling through TGF-β and TNF-α expression. Therefore, STAT3 decoy ODN is expected to treat liver injury as a direct STAT3 inhibitor.

One limitation of our study is that we did not perform an electrophoretic mobility shift assay (EMSA) to directly confirm the binding interaction between STAT3 decoy ODN and STAT3. This assay would be valuable for elucidating the mechanism of action, and we plan to perform it in future studies. Another limitation is that we did not use a scrambled ODN control in this study. Scrambled ODN (randomized nucleotides with the same backbone) help rule out non-specific effects. We acknowledge the potential for non-specific effects despite STAT3 decoy ODN’s sequence specificity. Therefore, future studies incorporating a scrambled ODN control will provide even stronger evidence for its sequence-specific efficacy.

In summary, STAT3 decoy ODN were successfully introduced into the liver of DDC-fed mice. This study demonstrated the anti-inflammatory and anti-fibrotic effects of STAT3 decoy ODN in suppressing STAT3 activation in hepatocytes and mouse models of liver injury. STAT3 decoy ODN inhibited STAT3 activation in hepatocytes, thereby reducing the levels of inflammatory and fibrotic factors. In conclusion, STAT3 decoy ODN demonstrated promising therapeutic effects in hepatocytes and mouse models of liver injury by suppressing fibrosis and inflammation and may be used in developing new treatment strategies. To further verify the therapeutic effectiveness of STAT3 decoy ODN, STAT3 knockout or knockdown cells or animal models can be used to confirm the role of STAT3 in inflammation and fibrosis. In addition, STAT3 decoy ODN is speculated to alleviate the toxic effects of drugs such as lipopolysaccharide-induced inflammation and fibrosis in cells. 

## 4. Materials and Methods

### 4.1. Decoy ODN Synthesis

High-performance liquid chromatography-purified DNA oligos were custom-made by Macrogen (Seoul, Republic of Korea). The sequences of STAT3 decoy ODN used were as follows (STAT3 binding sequences are underlined):

Human STAT3 decoy ODN: 

5′-CTTAAGCTAAATGCCCTTTACAAAAGTAAAGGGCATTTAG-3′

Mouse STAT3 decoy ODN: 

5′-CTTAAGGCGAAGGACCTTCGAAAACGAAGGTCCTTCGC-3′

These DNA oligos were annealed from 80 to 25 °C for 6 h. Both decoy ODN were predicted to be stem-ring-type. T4 DNA ligase (Takara Bio Inc., Shiga, Japan) was added at 16 °C for 16 h to ligate ring-type decoy ODN. FITC-labeled decoy ODN were created using Label IT^®^ Nucleic Acid Labeling Kit (Mirus Bio Corp., Madison, WI, USA).

### 4.2. Cell Culture

Human liver hepatocellular carcinoma cell lines, HepG2 (ATCC, Manassas, VA, USA) and HuH-7 (JCRB cell bank, Osaka, Japan), were cultured in Dulbecco’s Modified Eagle Medium comprising 10% fetal bovine serum and 1% penicillin–streptomycin at 37 °C in a humidified atmosphere with 5% CO_2_. The working concentrations of penicillin and streptomycin were 100 U/mL and 100 μg/mL, respectively. For transfection, serum-free Opti-MEM (Gibco, Grand Island, NY, USA) was mixed with Lipofectamine 2000 (Invitrogen, 11668-027, Waltham, MA, USA) and STAT3 decoy ODN. All transfections were performed using a standard reverse transfection assay.

### 4.3. Cell Viability Assay

Cell viability was measured using a cell counting kit-8 (CCK-8) (APExBIO, Houston, TX, USA). HepG2 cells were seeded at $1\times 10^{5}$ and HuH-7 cells were seeded at $2\times 10^{4}$ in 96-well plates for 24 h. CCK-8 reagent was added to each well at the end of incubation, followed by incubation at 37 °C for 2 h. The optical density was recorded at 480 nm using a microplate reader (Molecular Devices, San Jose, CA, USA).

### 4.4. Animal Model

Male C57BL/6 mice (6-week-old, 20–25 g; Samtako, Osan, Republic of Korea) were fed a diet containing 0.1% DDC for four weeks, housed under a 12 h light/dark cycle, and had access to water and diet ad libitum. Mice were randomly divided into three groups: (1) untreated (naïve); (2) vehicle-treated with 0.1% DDC-supplemented diet (DDC + Veh); and (3) STAT3 decoy ODN-treated with 0.1% DDC-supplemented diet (DDC + STAT3). STAT3 decoy ODN (20 μg) were injected twice weekly through the mouse tail vein using an in vivo gene delivery system (Mirus Bio Corp., Madison, WI, USA). After four weeks of treatment, mice were euthanized via intraocular vein blood collection after anesthesia. Their livers were then removed for further analysis. All experimental protocols were approved by the Institutional Animal Care and Use Committee of Daegu Catholic University Medical Center (approval number: DCIAFCR-220715-15-Y) following the Institutional Guidelines for Animal Research criteria.

### 4.5. Histopathological Investigation

The livers were fixed in a 10% formalin solution and embedded in paraffin for conventional light microscopy. According to standard protocols, the blocks were cut into 4 μm-thick sections and stained with H&E and Masson’s trichrome. All slides were examined using a Pannoramic MIDI slide scanner (3DHISTECH Ltd., Budapest, Hungary).

### 4.6. Immunohistochemical Staining

Xylene was used to deparaffinize the embedded tissue sections. The sections were dehydrated in progressively reducing ethanol concentrations and then treated with 3% hydrogen peroxidase in methanol for 10 min to prevent endogenous peroxidase activity. The liver tissue sections were placed in 10 mM sodium citrate buffer (pH = 6.0) for 5 min at 95 °C. The final step was repeated using a new 10 mM sodium citrate solution (pH 6.0). The sections were allowed to remain in the same solution while cooling for 20 min and then rinsed in phosphate-buffered saline. The tissue sections were incubated with a primary antibody such as E-cadherin (CST, Danvers, MA, USA, #3195S, 1:200), COL1A1 (Santa Cruz, Dallas, TX, USA, #sc8784, 1:200), F4/80 (Santa Cruz, #sc377009, 1:200), and TNF-α (Abcam, Cambridge, UK, ab1793, 1:200) for 1 h at 37 °C. The signal was visualized using EnVision System (DAKO, Agilent, Mississauga, ON, Canada) for 30 min at 37 °C. 3,3-diaminobenzidine tetrahydrochloride was used as a staining reagent, and hematoxylin was used as a counterstain. All slides were examined under the Pannoramic^®^ MIDI slide scanner and analyzed using Image J software (version 2.3.0).

### 4.7. Western Blot Analysis

HepG2 cells were seeded at $5\times 10^{6}$, and HuH-7 cells were seeded at $8\times 10^{5}$ in 100 mm dish. Both cells were treated with vehicle or transfected with STAT3 decoy ODN reaching 70~80% confluence, and scrapped 24 h after treatment or transfection for protein analysis. Liver tissue (100 mg) or cultured cells were homogenized in 1 mL of NP-40 lysis buffer supplemented with protease and phosphatase inhibitors (GenDEPOT, Baker, TX, USA). The protein concentration was determined using the BCA Protein Assay Kit (Thermo Fisher Scientific, Waltham, MA, USA). Total proteins (10–20 μg) were boiled for 10 min, loaded onto 10% homemade sodium dodecyl sulfate-polyacrylamide gel, and transferred to ImmunoBlot PVDF membrane (GE Healthcare, Chicago, IL, USA). The membrane was blocked for 1 h at room temperature in 5% skim milk and incubated with primary antibodies (1:1000 dilution) overnight at 4 °C. Horseradish peroxidase-conjugated secondary antibodies (1:2000 dilution) were used. The signal was analyzed by immunoblotting using an enhanced chemiluminescence solution (Thermo Fisher Scientific). The signal intensity was detected using an image analyzer (ChemiDoc™XRS+, Bio-Rad Laboratories, Hercules, CA, USA) and quantified using ImageJ software (version 2.3.0). The primary antibodies used in this study were anti-GAPDH (CST, #2118), anti-p-STAT3 (CST, #9145), anti-STAT3 (CST, #91390, anti-COX2 (CST, #12282), anti-TNF-α (Abcam, #ab6671), anti-COL1A1 (Santa Cruz, #sc8784), anti-Vimentin (BD, #550513), anti-TFG-β (Abcam, #ab215715), and anti-IFN-γ (Abcam, #ab9657).

### 4.8. RNA Purification and qPCR Analysis

Total RNA was isolated from snap-frozen liver tissues or 12-well plate-cultured cells using TRIzol Reagent (Thermo Fisher Scientific), and cDNA was prepared using a reverse transcription kit (Takara Bio Inc.). Hepatic gene expression was determined via qPCR using AccuPower^®^ 2X GreenStar ^®^ qPCR Master Mix (ROX dye) (Bionner, Daejeon, Republic of Korea). mRNA levels were normalized to those of *GAPDH*. qPCR primer sequences used in the study are listed in Table 1.

### 4.9. Statistical Analysis

The sample size for the experiments was empirically determined based on preliminary experiments to ensure appropriate statistical power. Values are expressed as the mean ± standard error of the mean (SEM) or standard deviation (SD), and error bars for results were derived from biological or technical replicates. Statistical analysis was performed using one-way ANOVA to compare the differences between three groups (in vivo) and Student’s *t*-test to compare the differences between two groups. A *p*-value of < 0.05 was considered statistically significant.

## Figures and Tables

**Table 1:** qPCR primer sequences used in this study.

Primer	Sequence (5′-3′)
hGAPDH_Fwd	GTCTCCTCTGACTTCAACAGCG
hGAPDH_Rvs	ACCACCCTGTTGCTGTAGCCAA
hMMP2_Fwd	AGCGAGTGGATGCCGCCTTTAA
hMMP2_Rvs	CATTCCAGGCATCTGCGATGAG
hMMP9_Fwd	GCCACTACTGTGCCTTTGAGTC
hMMP9_Rvs	CCCTCAGAGAATCGCCAGTACT
hACTA2_Fwd	CTATGCCTCTGGACGCACAACT
hACTA2_Rvs	CAGATCCAGACGCATGATGGCA
hTGF-β1_Fwd	TACCTGAACCCGTGTTGCTCTC
hTGF-β1_Rvs	GTTGCTGAGGTATCGCCAGGAA
hTIMP1_Fwd	GGAGAGTGTCTGCGGATACTTC
hTIMP1_Rvs	GCAGGTAGTGATGTGCAAGAGTC
hTIMP3_Fwd	TACCGAGGCTTCACCAAGATGC
hTIMP3_Rvs	CATCTTGCCATCATAGACGCGAC
hTNF-α_Fwd	CTCTTCTGCCTGCTGCACTTTG
hTNF-α_Rvs	ATGGGCTACAGGCTTGTCACTC
hc-Myc_Fwd	CCTGGTGCTCCATGAGGAGAC
hc-Myc_Rvs	CAGACTCTGACCTTTTGCCAGG
hSnail_Fwd	TTACACCTTTGCATACAGAACCC
hSnail_Rvs	TTTACGATTACACCCAGACTGC
hSAA1_Fwd	TCGTTCCTTGGCGAGGCTTTTG
hSAA1_Rvs	AGGTCCCCTTTTGGCAGCATCA
hCOL1A1_Fwd	GATTCCCTGGACCTAAAGGTGC
hCOL1A1_Rvs	AGCCTCTCCATCTTTGCCAGCA
hp21_Fwd	AGGTGGACCTGGAGACTCTCAG
hp21_Rvs	TCCTCTTGGAGAAGATCAGCCG

## Data Availability

Data are contained within the article or Supplementary Material.

## Supplementary Materials

The following supporting information can be downloaded at: https://www.mdpi.com/article/10.3390/molecules29030593/s1, Graphical abstract; Figure S1: Western blot raw image of Figure 2b; Figure S2: Western blot raw image of Figure 3b; Figure S3: Western blot raw image of Figure 5c.
