# Supplementary material for: STAT3 Decoy Oligodeoxynucleotides Suppress Liver Inflammation and Fibrosis in Liver Cancer Cells and a DDC-Induced Liver Injury Mouse Model
Source: Molecules. 2024 Jan 25;29(3):593. doi: 10.3390/molecules29030593 (PMC10856653; doi:10.3390/molecules29030593)
Supplement: Supplementary file 1 [file molecules-29-00593-s001.zip › molecules-2759699-supplementary.pdf]

Supplementary Figure S1

a

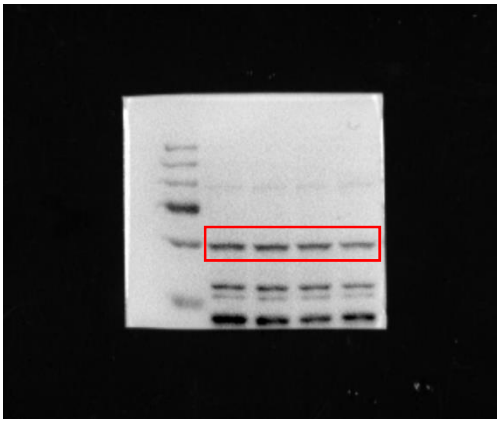

b

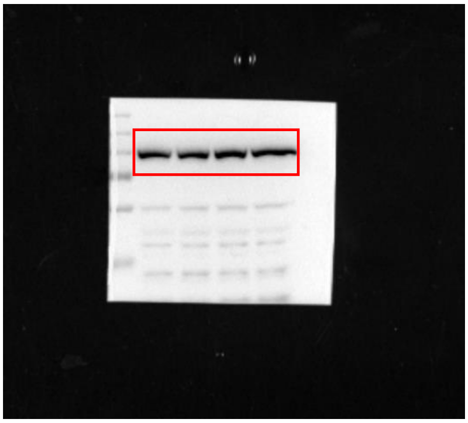

c

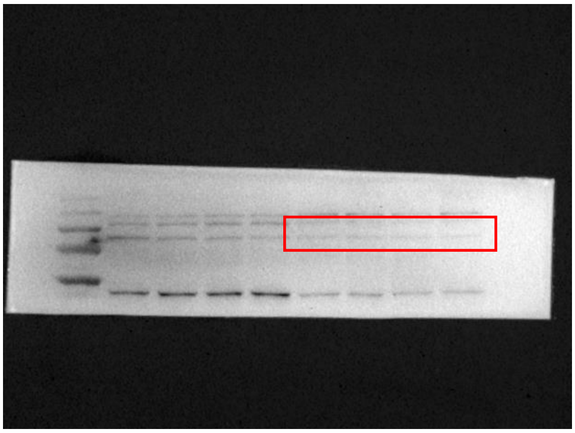

d

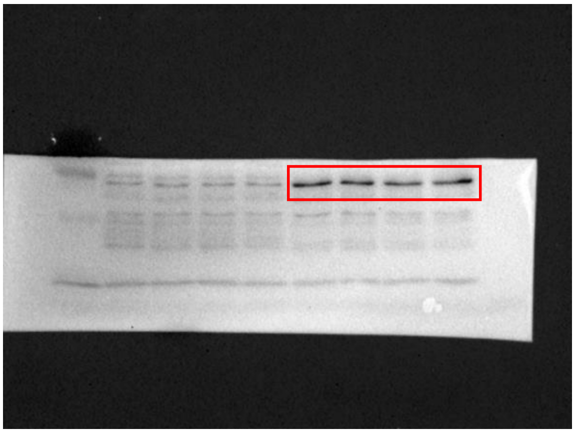

e

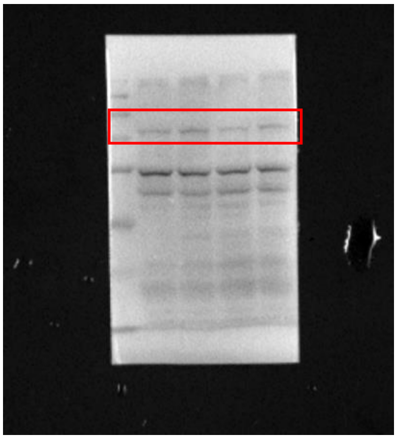

f

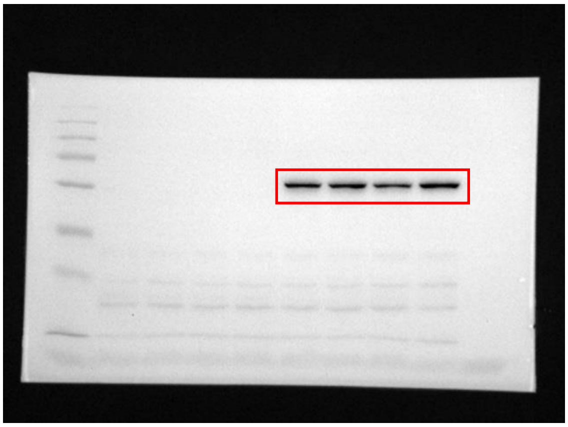

g

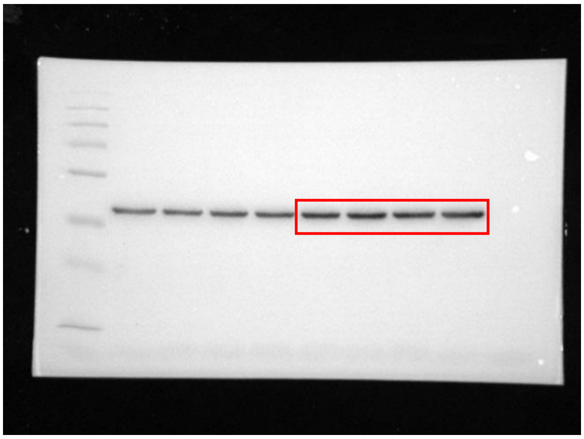

Supplementary Figure S2

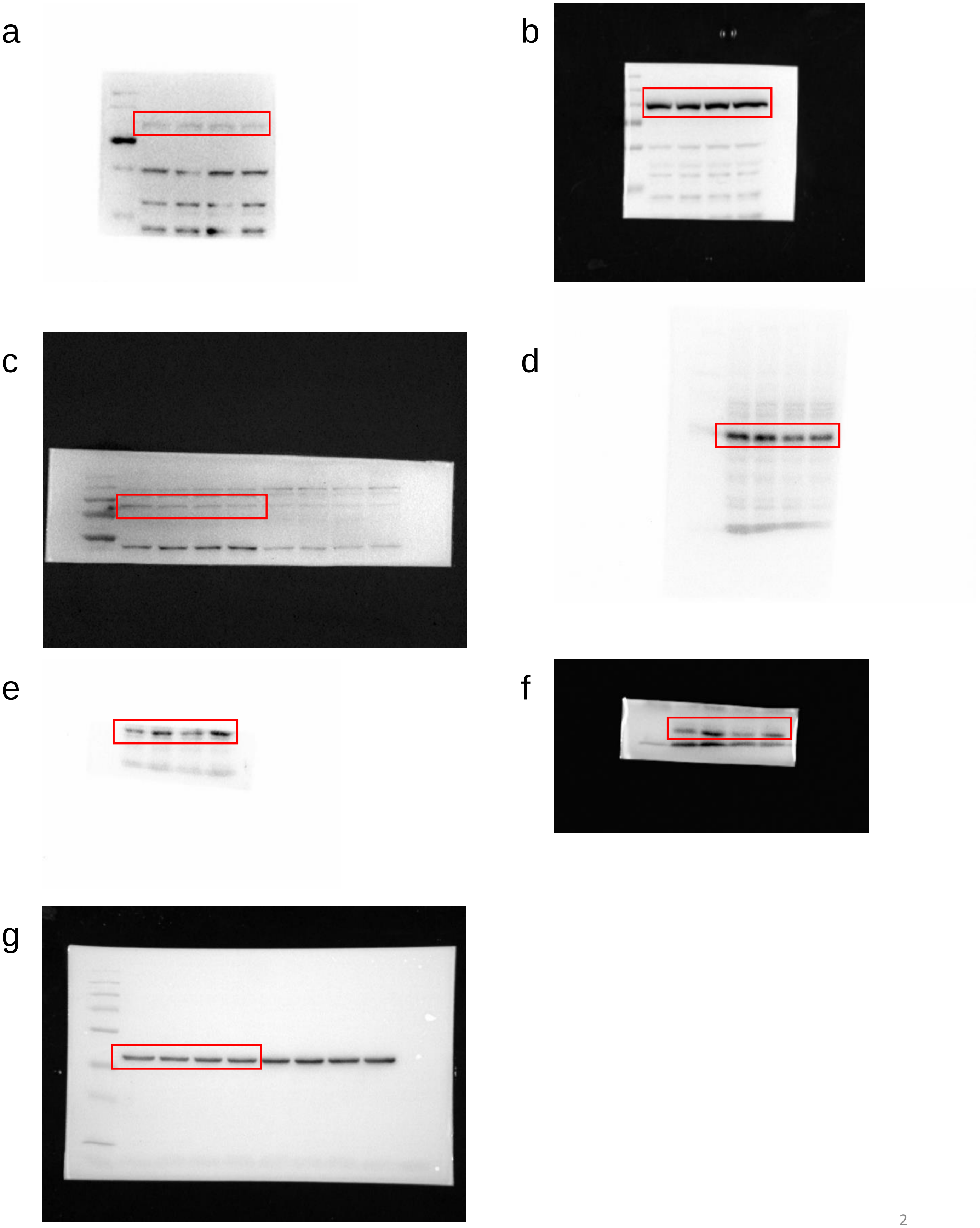

Supplementary Figure S3

a

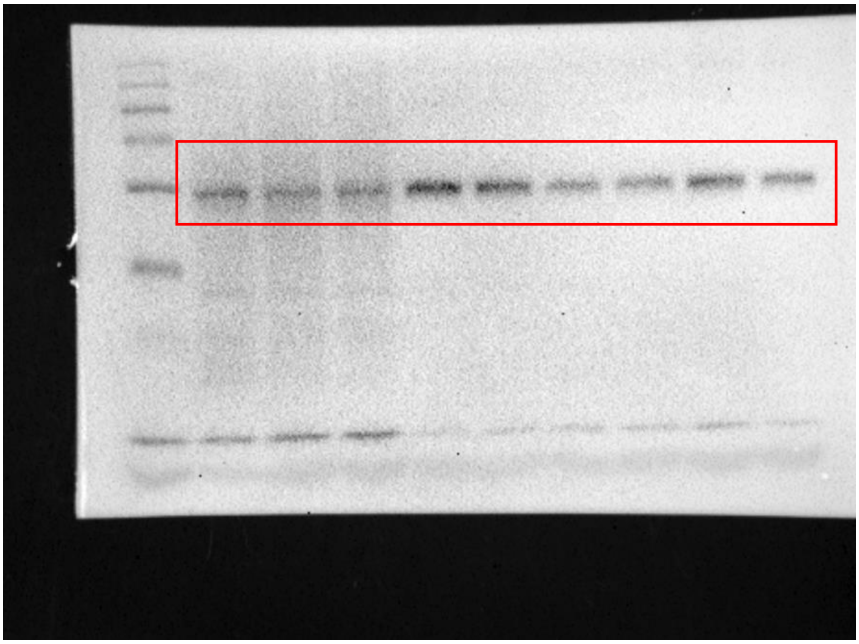

b

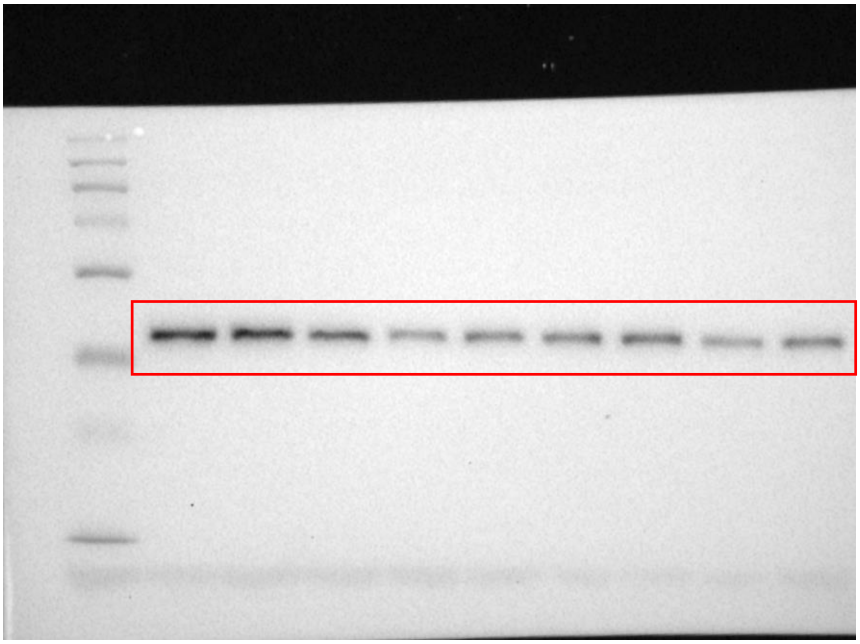

Supplementary Figure legends

Supplementary Figure S1. Western blot raw image of Figure 2b. phospho-STAT3 (a), STAT3 (b), COX2; four on the right (c), TNF-α; four on the right (d), COL1A1 (e), Vimentin; four on the right (f), GAPDH; four on the right (g).

Supplementary Figure S2. Western blot raw image of Figure 3b. phospho-STAT3 (a), STAT3 (b), COX2; four on the left (c), TGF-β (d), TNF-α (e), IFN-γ (f), GAPDH; four on the left (g).

Supplementary Figure S3. Western blot raw image of Figure 5c. TGF-β (a), GAPDH (b).
